# Deletion of *Rapgef6*, a candidate schizophrenia susceptibility gene, disrupts amygdala function in mice

**DOI:** 10.1038/tp.2015.75

**Published:** 2015-06-09

**Authors:** R J Levy, M Kvajo, Y Li, E Tsvetkov, W Dong, Y Yoshikawa, T Kataoka, V Y Bolshakov, M Karayiorgou, J A Gogos

**Affiliations:** 1 Department of Neuroscience, Columbia University, New York, NY, USA; 2 Department of Physiology and Cellular Biology, Columbia University, New York, NY, USA; 3 Department of Psychiatry, Columbia University, New York, NY, USA; 4 Department of Psychiatry, McLean Hospital, Harvard Medical School, Belmont, MA, USA; 5 Department of Otolaryngology, Loma Linda University, Loma Linda, CA, USA; 6 Division of Molecular Biology, Department of Biochemistry and Molecular Biology, Kobe University Graduate School of Medicine, Kobe, Japan

## Abstract

In human genetic studies of schizophrenia, we uncovered copy-number variants in *RAPGEF6* and *RAPGEF2* genes. To discern the effects of *RAPGEF6* deletion in humans, we investigated the behavior and neural functions of a mouse lacking *Rapgef6*. *Rapgef6* deletion resulted in impaired amygdala function measured as reduced fear conditioning and anxiolysis. Hippocampal-dependent spatial memory and prefrontal cortex-dependent working memory tasks were intact. Neural activation measured by cFOS phosphorylation demonstrated a reduction in hippocampal and amygdala activation after fear conditioning, while neural morphology assessment uncovered reduced spine density and primary dendrite number in pyramidal neurons of the CA3 hippocampal region of knockout mice. Electrophysiological analysis showed enhanced long-term potentiation at cortico–amygdala synapses. *Rapgef6* deletion mice were most impaired in hippocampal and amygdalar function, brain regions implicated in schizophrenia pathophysiology. The results provide a deeper understanding of the role of the amygdala in schizophrenia and suggest that *RAPGEF6* may be a novel therapeutic target in schizophrenia.

## Introduction

Recent genetic advances demonstrated that there is a shared genetic diathesis among neuropsychiatric disorders.^1^ This common genetic etiology implies there may be a shared pathophysiology among these disorders. Genetic data from a variety of schizophrenia studies converge onto the *RAPGEF6* locus. We discovered copy-number variants involving *RAPGEF2* and *RAPGEF6* as well as a nonsynonymous mutation in *RAPGEF2* within a cohort of patients with schizophrenia.^2, 3, 4^ The *RAPGEF6* copy-number variant was a heterozygous (HET) *de novo* deletion of exons 2–11 and thus predicted to cause a loss of function. Earlier studies also suggested a possible role for Rapgef6 in mental illness. *RAPGEF6* was part of a large deletion associated with schizophrenia and mental retardation in a single patient,^5^ and the 5q31.1 locus around this gene is the fourth most important schizophrenia linkage peak.^6, 7, 8, 9, 10, 11, 12, 13^ Finally, single-nucleotide polymorphism genotyping demonstrated association with a block of linkage disequilibrium including *RAPGEF6.*^14, 15, 16^ Considering overlap with other psychiatric diseases, anxiety and depression are associated with single-nucleotide polymorphisms in *RAPGEF3* (ref. 17) while *RAPGEF4* single-nucleotide polymorphisms were associated with autism risk.^18^

Functionally, *RAPGEF6* is a guanine exchange factor, which activates GTPases Rap1 and Rap2 by exchanging GDP for GTP.^19^ Downstream of *Rapgef6*, Rap1 interacts with JamA, Afadin, Bag3, Riam and RapL to modulate cadherins and integrins that mediate adhesion to other cells and the extracellular matrix.^20, 21, 22, 23^ These *Rapgef6* pathways were demonstrated to affect adherens junctions between cells, integrin junctions to the matrix, actin organization and migration in epithelial and lymphatic cells. To date, little is known about the function of *Rapgef6* in neurons except that knocking it down reduces neurite length downstream of NRF-1.^24^ Initial assessment of a *Rapgef6* knockout mouse uncovered splenomegaly and diminished lymphocyte adhesion via integrins.^20^

Other Rapgefs and Raps have striking neural phenotypes when deleted and contribute to neural guidance, morphology and neuronal functions (Supplementary Table 1). As *Rapgef6* is both a plausible functional and genetic candidate for schizophrenia risk, we performed a comprehensive analysis of mice lacking *Rapgef6* to uncover its role in synaptic plasticity and behavioral paradigms dependent on learning, as well as neurite architecture. We discovered that *Rapgef6* deletion had a specific and circumscribed phenotype. Rapgef6 impacts amygdala-dependent fear learning, as well as neural activation in the hippocampus and amygdala during fear conditioning. At the functional level, *Rapgef6* affects cortico–amygdala long-term potentiation (LTP) and CA3 hippocampal spine density.

## Materials and methods

### Western blotting

Mouse brain regions were excised and crude synaptosomal preparations were made by homogenizing in buffer containing 5 mM Hepes/10% sucrose (pH 7.5). *Rapgef6* protein is predicted to be 177.9 kDa. Antiserum was generated in rabbits against the C-terminal synthetic peptide GLEPRDTTDPVYKTVTSSTD located at amino acids 1474–1494.^20^ Primary rabbit anti-*Rapgef6* antibody was used at 1:100 (see Supplementary Information for more details of Materials and methods).

### Mouse knockout

All animal procedures were performed according to protocols approved by the Institutional Animal Care and Use Committees established by Columbia University under federal and state regulations. *Rapgef6* knockout animals were generated by the Kataoka laboratory and shared via RIKEN.^20^ Briefly, exon 21 was floxed, transfected into oocytes and bred, then *Rapgef6*^*+/flox*^ mice were bred with *CAG-Cre* mice to yield *Rapgef6*^*+/*^^−^ animals lacking the GEF catalytic domain. Mice were backcrossed three generations to C57/Bl6. Genotyping was performed as previously described.^20^ All experiments were performed on age-matched sets of male littermates from HET × HET breedings. We did not perform a power calculation as we could not predict an *a priori* effect size. Sample sizes were estimated on the basis of acceptable standards found in our prior published work and similar reports by other investigators. Animals or cells were not excluded from experiments unless there was technical failure (culture contamination, inability to confirm genotype, failure of immunocytochemistry protocol). Animals and cells were not randomized because they were instead defined by genotype and then litter- and age-matched by genotype. The experimenters remained masked to genotype while performing all experiments, analyzing images and analyzing data. A third party re-coded and grouped the animals or cells to maintain masking. After all analyses, tissue was re-genotyped for confirmation.

### Open field

Animals were habituated for 30–60 min, then monitored in transparent open-field chambers (25 cm^2^) with infrared motion detection beams (Coulbourn Instruments, Whitehall, PA, USA) for 1 h on day 1 and 30 min on day 2. Locomotion in the horizontal and vertical planes was tracked via laser beam breaks and the data analyzed in TruScan software as distance traveled or time in the margins (<2.5 beams from wall) and center (rest of chamber).

### Novel object recognition

For two days, animals were habituated in an empty cage for 10 min. The mice were then exposed to two identical green objects for 5 min. One hour later, they were exposed to one green object and one yellow object, balanced across genotypes for left and right sides. Three hours later, the novel yellow object was replaced again with a red object. Videotape was hand-scored by a masked observer for time spent in direct contact with each object over the 5-min period. Percent time per novel object was calculated as (total time investigating novel object)/(total time investigating both objects).

### Morris water maze

Male mice (3–4 months old) were tested as previously described using AnyMaze software (Stoelting, Wood Dale, IL, USA).^25, 26^

### T-maze

Mice were habituated to the maze for 10 min on 3 days, then 2 days of forced-alternation training. In choice training, each mouse was timed until it reached the empty food dish of the open arm, then returned to the start. Both arms were opened and the choice of arms (goal or non-goal) and time to the food dish were recorded. There was a 40 s intertrial delay with 10 daily repetitions. Training continued until each mouse reached criterion of 7 out of 10 correct choices on three consecutive days. Next, animals began 3 days of working memory testing. The intratrial delay was increased to 10, 20 or 30s with four trials of each delay time pseudorandomized across the session.

### Fear conditioning

Conditioning was performed as previously described with the following modifications.^27^ The testing occurred on 2 days, with conditioning on the first day and cued evaluation 24 hours later, followed by contextual evaluation 2 h subsequently. The conditioned tone was 30 s at 85 dB and 2 kHz, which co-terminated with a 1 s 0.7 mA shock; this pairing was delivered two times 60 s apart. During cued evaluation, the tone began 180 s into the trial and persisted for 180 s. Odors used to enhance novelty were lemon during the first two trials and vanilla during the novel context trial. Digital video was analyzed using FreezeFrame software (Coulbourn).

### cFOS activation after fear conditioning

Fear conditioning was executed as described above. Following conditioning assessment, animals were left in the chambers for 90–120 min, then perfused with phosphate-buffered saline and 4% PFA. After overnight post-fixation, brains were sliced 60 μm thick and every other section was stained with rabbit anti-cFOS 1:5000 (Calbiochem, San Diego, CA, USA), mouse anti-NeuN 1:300 (EMB Millipore, Billerica, MA, USA) and TOPRO 1:2500.

These slides were viewed at × 20 on a confocal microscope to permit manual counting of cFOS+ cells in the dentate gyrus, CA3 and CA1 subregions of the hippocampus as well as the lateral, basolateral and central nuclei of the amygdala. Regions of interest were defined using a mouse atlas to set anatomic boundaries and analyzed from Bregma −1.3 to −1.9 with TOPRO staining providing anatomic demarcations.^28^ The dentate gyrus is a clearly demarcated structure. Dorsal CA1 hippocampus was defined from the end of the blades of the dentate gyrus to the end of the mossy fiber pathway. Dorsal CA3 began at the end of the mossy fiber pathway and terminated at the midline. The basolateral nucleus of the amygdala was defined as the lower half of the region within the forking of the external capsule while the lateral nucleus was the upper half. The central nucleus was medial to the lateral/basolateral nucleus.

### *In vivo* dendritic morphology analysis

*Rapgef6* HET animals were crossed with the Thy1-M-GFP mouse line, which expresses GFP in a mosaic fashion in pyramidal neurons.^29^ Ten- to 12-week-old male wild-type (WT), HET and homozygous (HOM) littermates from *Rapgef6*^+/^^−^ × *Rapgef6*^+/^^−^ GFP^+^ matings were, perfused with phosphate-buffered saline and 4% PFA followed by overnight fixation in 4% PFA. The brains were sliced in 100 μm sections, washed 3 × 5 min in phosphate-buffered saline and stained with TOPRO 1:2500 for 10 min.

Regions of interest were defined using a mouse brain atlas to set anatomic boundaries.^28^ The dorsal hippocampus CA3 was defined by the flexure of the mossy fiber pathway to its end. Dorsal CA1 began at the end of the mossy fiber pathway and terminated at the midline. Both of these were imaged from Bregma coordinates −1.3 to −1.9. Due to dense GFP labeling obscuring the apical dendritic arbors, only basal dendrites could be analyzed. Although these are not the synapses of the classical trisynaptic hippocampal pathway, we assumed there would be global changes. Medial prefrontal cortex was imaged from Bregma +2 to +1.5, with pre- and infralimbic subregions defined as the upper and lower halves of the tissue medial to the forceps minor of the corpus callosum. The lateral/basolateral nuclei of the amygdala were defined as the region within the forking of the external capsule from Bregma −1.1 to −1.9.

### Morphology imaging and analysis

Sections were imaged on a confocal microscope (Carl Zeiss, Oberkochen, Germany) at × 20 zoom for neurites and × 63 zoom for spines. For spines, images were taken after the first primary dendrite branch point. A maximum intensity projection image was generated from the three-dimensional image stack. Images were loaded into the NeuronJ plug-in for ImageJ, where the neural processes were manually traced and labeled. For spine assessment, spines were manually counted and measured using LSM software (Carl Zeiss) if there was a visible neck connecting to the dendrite. Spine morphology was assessed according to head shape and neck measurements.^30^

For Sholl analysis, a macro in ImageJ generated concentric circles 50 μm apart, which was initiated over the center of the soma. Crossings of neurite tracings over circles were then manually counted.

### Electrophysiology

Slices of the amygdala (300 μm) were prepared from 3.5- to 4-month-old mice in cold cutting solution (see Supplementary Information for solution composition). After incubation for 50 min at room temperature, individual slices were transferred into the recording chamber, which was continuously perfused with recording solution. Whole-cell recordings of synaptic responses were obtained from principal neurons in the lateral nucleus of the amygdala under visual guidance (DIC/infrared optics) with an EPC-10 amplifier and Pulse v8.8 software (HEKA Elektronik, Lambrecht, Germany). Currents were filtered at 1 kHz and digitized at 5 kHz. Synaptic responses were evoked by stimulation of fibers in the external capsule (cortical input) or the internal capsule (thalamic input) by a concentric stimulation electrode.^31^ The excitatory postsynaptic current (EPSC) or excitatory postsynaptic potential (EPSP) amplitudes were measured as the difference between the mean amplitude during the pre-stimulus baseline and the mean amplitude over a 1–2- ms window at the response peak. LTP was induced and recorded in current-clamp mode. For the induction, 80 presynaptic stimuli were delivered to cortical input at 2 Hz, paired with action potentials evoked in a recorded postsynaptic neuron with 4–8 ms delay from the onset of each EPSP.^32^ Summary LTP graphs were constructed by normalizing data in 60-s epochs to the mean value of the baseline EPSP. The LTP magnitude was estimated in a time window of 5 min at 40 min after the induction. The NMDA/AMPA amplitude ratio was calculated by dividing the amplitude of the NMDA receptor component (measured at +40 mV at 40 ms after the peak of AMPA receptor EPSCs at −70 mV) by the peak AMPA receptor EPSC at −70 mV. mEPSCs were recorded in the presence of 1 μM tetrodotoxin and analyzed with the Mini Analysis Program v6.0.7 (Synaptosoft, Decatur, GA, USA).

### Statistics

Statistical analyses for behavioral and morphologic data were analyzed as analysis of variance (ANOVA, for genotype effects) or repeated measures ANOVA (for genotype × time effects) followed by Bonferroni-corrected *post hoc* testing in Prism (GraphPad, La Jolla, CA, USA). For electrophysiology, data were analyzed as two-way ANOVA (for input–output curves and paired-pulse facilitation) or unpaired *t*-test (for mEPSCs, LTP and NMDA/AMPA amplitude ratio). Formal normality testing was not performed. Visual inspection of individual data points revealed normally distributed data with no obvious deviations. Each analysis has variance estimated and reported or graphed as ±s.e.m. Variances were not significantly different between the groups. Figures are shown as mean±s.e.m. and F and/or *P*-values are reported with *P*<0.05 as the threshold for significance.

## Results

### *Rapgef6* is expressed in the amygdala and the hippocampus

The Allen Brain Atlas (www.brain-map.org) predicted that *Rapgef6* mRNA is expressed at low levels throughout the mouse cortex, with higher expression in the hippocampus particularly within CA3 and in the amygdala, areas implicated in neuropsychiatric disorders.

Western blot analysis using a previously published antibody against *Rapgef6* (ref. 20) confirmed *Rapgef6* protein expression in the amygdala and hippocampus as well as the prefrontal cortex (Supplementary Figure 1A). *Rapgef6* protein was also readily detected in crude synaptosomal fractions, suggesting synaptic localization. Within the hippocampus, *Rapgef6* was found in the dentate gyrus, CA3 and CA1 (Supplementary Figure 1B). Notably, no protein was detected by western blot in HOM mice, indicating successful knockout, as had been previously published.^20^

### *Rapgef6* knockout impairs anxiety-like behavior and fear conditioning

HET mice were included in all experiments as this recapitulated the human mutation, while HOM knockout mice were predicted to have a more severe phenotype. Cresyl violet staining of brains demonstrated that *Rapgef6* HOM and HET animals were not grossly different from WT littermates in their neural architecture. Unlike *Rapgef2* knockout mice, no heterotopias, aberrant white matter tracts, or absence of brain regions were noted (data not shown).^33^ A large battery of behavior paradigms were tested first to identify domains or brain regions affected by *Rapgef6* deletion.

The open field tests locomotion as well as anxiety-like behavior regarding avoidance of the arena center.^34^ Hyperlocomotion is thought to be analogous to dopamine-sensitive psychomotor agitation in patients with schizophrenia.^35, 36^ In the open field, HET and HOM mice demonstrated increased distance in the center of the arena and increased rearing (Supplementary Figure 2A,B; *n*=26 WT, 24 HET, 16 HOM mice, ANOVA, center distance: F_(2,63)_=4.36, *P*=0.017; rearing: F_(2,63)_=9.22, *P*=0.0003; *P*<0.05 on Bonferroni *post hoc* testing for all inter-genotype comparisons). Total distance traveled was affected by genotype and increased in HET mice over WT littermates with a trend toward increased distance in HOM mice (data not shown; *n*=26 WT, 24 HET, 16 HOM mice, ANOVA, F_(2,63)_=3.75, *P*=0.029, Bonferroni *post hoc P*<0.05).

Hippocampal function can be measured by spatial memory tasks such as the Morris water maze and novel object recognition,^37, 38^ while prefrontal function underlies working memory performance as assessed by the T-maze test.^39, 40^ Many neuropsychiatric diseases, especially schizophrenia, have profound cognitive deficits and animal cognitive performance, including maze learning, is considered a valid assessment of this disease component.^41^

*Rapgef6* knockout mice did not differ from WT on a variety of memory tasks. HOM mice were not significantly worse on performance of hippocampal-based spatial memory tasks such as the Morris water maze because all animals learned to find (data not shown) and prefer the platform quadrant (Supplementary Figure 2C, *n*=14 WT, 12 HET, 7 HOM mice, ANOVA, F_(11,118)_=8.15, *P*<0.001, Bonferroni *post hoc* *P*<0.05 for comparisons to NW quadrant). There was also no effect of genotype on the ability to recognize novel objects, another hippocampal spatial memory task (Supplementary Figure 2D, *n*=12 WT, 9 HET, 9 HOM mice, ANOVA, F_(2,30)_=0.57, *P*=0.57; one sample *t*-test comparison against 50% with df=11; WT: *t*=4.83, *P*=0.0005; HET: *t*=2.48, *P*=0.03; HOM: *t*=3.14, *P*=0.01). Finally, deleting *Rapgef6* did not alter performance on a prefrontal cortex-dependent T-maze test of working memory (Supplementary Figure 2E, *n*=13 WT, 16 HET, 16 HOM mice, ANOVA, effect of genotype F_(2,68)_=0.25, *P*=0.78) though all animals did learn the task (ANOVA, effect of intratrial delay F_(2,68)_=5.40, *P*=0.007).

The most striking cognitive findings concerned the fear-conditioning paradigm. Fear conditioning is a classical conditioning paradigm that relies primarily on hippocampal and amygdala function for contextual learning and amygdala function for cued fear learning.^42, 43^ Fear-conditioning abnormalities in rodents are considered most analogous to human anxiety disorders (that is, generalized anxiety disorder and posttraumatic stress disorder), but may also model the negative symptoms of schizophrenia such as affective flattening.^44^

**Figure 1:**
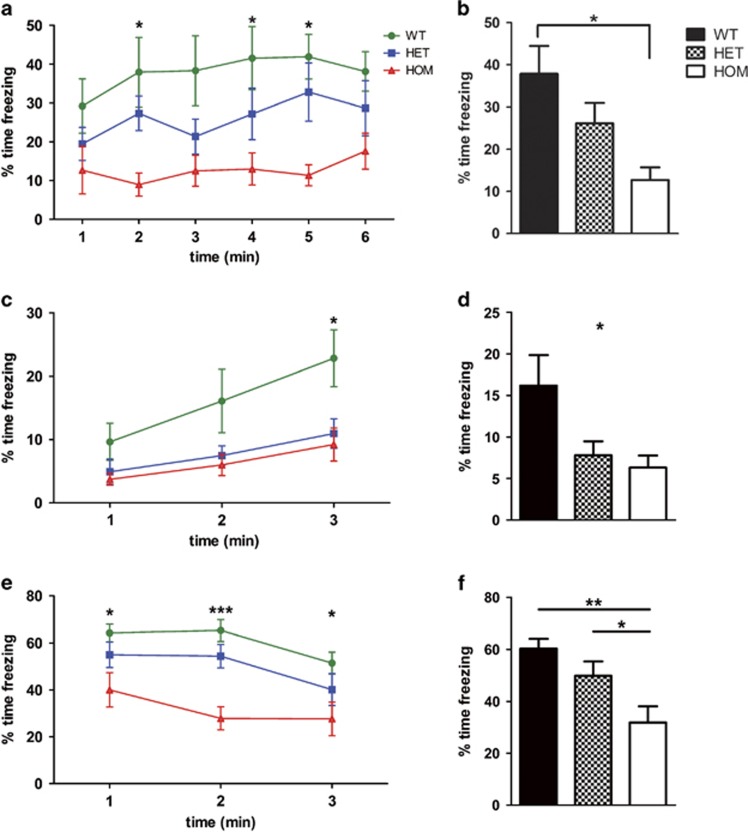
*Rapgef6* deletion impairs contextual and cued fear conditioning, implying amygdala dysfunction. (**a**) Contextual fear conditioning had a significant effect of genotype (*P*=0.016) but not test time (*P*=0.09) with HOM mice freezing less in the original context at the second, fourth and fifth minutes (*P*<0.05). (**b**) Averaged contextual fear also had a significant effect of genotype (*P*=0.016), with HOM mice freezing significantly less than WT (*P*<0.05). (**c**) In the novel context, there was a significant effect of genotype (*P*=0.03) with HET and HOM mice freezing less than WT in the final minute (*P*<0.05). (**d**) Averaged novel context was also significantly affected by genotype (*P*=0.03) with no *post hoc* comparisons significant. (**e**) Cued fear conditioning had a significant effect of genotype (*P*=0.003). HOM mice froze less than WT at each time point (*P*<0.05). (**f**) Averaged cued fear was also significantly affected by genotype (*P*=0.003) with HOM mice freezing less than HET (*P*<0.05) and WT (*P*<0.01). *P<0.05, **P<0.01 and ***P<0.001. HET, heterozygous; HOM, homozygous; WT, wild type.

There were no significant differences between genotypes on baseline fear as measured by initial freezing response before tone-shock pairings or nociception as measured by freezing during the two delivered shocks (Supplementary Figure 3). HOM mice froze significantly less on contextual and cued fear conditioning, indicating widespread fear learning deficits. On contextual testing, HOM mice froze significantly less than WT littermates, suggesting impairment of the hippocampus and/or amygdala (Figure 1a, *n*=12 WT, 12 HET, 9 HOM mice for all fear experiments, repeated measures two-way ANOVA, effect of genotype, F_(2,145)_=4.78, *P*=0.016, Bonferroni *post hoc* *P*<0.05 for WT vs HOM at the second, fourth and fifth minute; Figure 1b, ANOVA, F_(2,29)_=4.78, *P*=0.016, Bonferroni *post hoc* *P*<0.05).

HET and HOM mice also froze less in the novel context before the tone to test cued fear conditioning, indicating less generalization of fear learning (Figure 1c, repeated measures two-way ANOVA, effect of genotype, F_(2,58)_=3.95, *P*=0.03, Bonferroni *post hoc* *P*<0.05 for last minute; Figure 1d, ANOVA, F_(2,29)_=3.95, *P*=0.03, no Bonferroni *post hoc* comparisons significant).

During cued conditioning testing, HOM mice froze less than HET and WT possibly implicating amygdala dysfunction. There was a significant effect of genotype (Figure 1e, repeated measures two-way ANOVA, effect of genotype, F_(2,29)_=6.95, *P*=0.003, Bonferroni *post hoc* *P*<0.05) and when data were averaged (Figure 1f, ANOVA, F_(2,29)_=6.95, *P*=0.003 Bonferroni *post hoc* HOM vs HET *P*<0.05 HOM vs WT *P*<0.01). Notably, auditory testing on a limited sample of WT and HOM mice did not find any significant deficits in audition after deletion of *Rapgef6*; therefore, hearing abnormalities do not account for the cued conditioning phenotype (Supplementary Figure 4).

Mouse behavior analysis demonstrated that *Rapgef6* deletion did not impact hippocampal-dependent spatial memory or prefrontal cortex-dependent working memory. Since fear conditioning was impaired, this deficit is likely due to amygdala dysfunction as the amygdala contributes to both contextual and cued conditioning. Finally, *Rapgef6* mice were mildly hyperactive by measurements of locomotion and rearing and had reduced anxiety-like behavior.

### *Rapgef6* deletion has limited impact on dendritic morphology

To investigate whether *Rapgef6* deficiency affects the morphology of neurons in these structures, we analyzed dendrites of hippocampal and amygdala neurons by crossing knockout mice with a mosaic GFP reporter line as previously described in other neuropsychiatric disease models to analyze differences in neuroanatomy.^26, 45^

We analyzed basal dendritic arbors of neurons in CA3 and CA1 subregions of the hippocampus to correspond with spatial memory tasks. In CA3, there was no effect of genotype on total dendritic length (Supplementary Figure 5A, *N*=3 WT, 4 HET, 4 HOM mice for all the hippocampal morphology experiments, *n*=26 WT, 40 HET, 33 HOM neurons for all CA3 branching morphology experiments, ANOVA, F_(2,95)_=1.34, *P*=0.27), nor on the number of dendritic branches (Supplementary Figure 5B, ANOVA, F_(2,95)_=0.74, *P*=0.48). On Sholl analysis of CA3, there were no significant differences in crossings (data not shown).

At the next step of the hippocampal trisynaptic pathway in area CA1, there were no significant effects of genotype on morphology. Total basal dendritic length (Supplementary Figure 5C, *n*=18 WT, 22 HET, 21 HOM neurons for all CA1 branching experiments, ANOVA, F_(2,58)_=0.73, *P*=0.49) and number of dendritic branches (Supplementary Figure 5D, ANOVA, F_(2,58)_=1.31, *P*=0.31) were all equivalent among genotypes. Sholl analysis of CA1 neurons did not yield any differences by genotype (data not shown).

CA3 basal dendritic spine density was significantly affected by genotype (Supplementary Figure 5E, *n*=32 WT, 34 HET, 29 HOM neurons, ANOVA, F_(2,87)_=5.29, *P*=0.007). HOM spine density was reduced nearly 20% relative to both WT and HET spine density (Bonferroni *post hoc* *P*<0.05 for both comparisons). In contrast, CA1 basal spine density did not differ among genotypes (Supplementary Figure 5F, *n*=26 WT, 37 HET, 35 HOM neurons, ANOVA, F_(2,95)_=1.91, *P*=0.15).

In the basolateral amygdala of these animals, the amygdala nucleus essential to fear processing,^46^ spine density was counted along the apical and basal dendritic arbors of pyramidal neurons. Neither apical nor basal dendritic spine density were affected by genotype (Supplementary Figure 5G, H; *N*=3 WT, 3 HET, 3 HOM mice for all the amygdala morphology experiments; apical: *n*=46 WT, 38 HET, 29 HOM neurons, ANOVA, F_(2,110)_=0.34, *P*=0.71; basal: *n*=121 WT, 104 HET, 101 HOM neurons, ANOVA, F_(2,323)_=1.15, *P*=0.46).

Finally, the pre- and infralimbic subregions of the medial prefrontal cortex layer V pyramidal neurons were analyzed for basal dendritic morphology. Basal dendritic length and number of dendritic branches were not significantly affected by genotype (data not shown).

To understand the mechanistic basis of the behavior deficits, we analyzed the corresponding brain regions. In the hippocampus subregion, CA3 spine density was reduced but no other hippocampal or medial prefrontal cortex measurements were affected, which was unsurprising as hippocampal spatial memory and cortical working memory were intact. Finally, though amygdala performance was impaired on fear conditioning, there were no changes in basolateral spine density in this region. Not all behavioral findings will correlate with anatomic changes; the genetic effects could lie at the level of molecular or synaptic alterations.

### *Rapgef6* knockout reduced hippocampal and amygdala activation by cFOS

We further analyzed the effects of *Rapgef6* deletion on neural activation. cFOS is an early component of the synaptic plasticity pathway and its staining pattern is a reliable measure of neural activation as the number of cFOS-positive neurons positively correlates with fear learning.^47, 48^ It has been previously demonstrated that cFOS expression is significantly upregulated in the basolateral, lateral and central amygdala and CA1 hippocampus within 90 min after fear conditioning, mirroring activation in these brain regions (Supplementary Figure 3E).^48, 49, 50^ To investigate the activation of the amygdala and hippocampus during fear conditioning, mice were trained with or without the unconditioned shocks, then cFOS expression was assessed. This comparison allows analysis of the effects of fear conditioning and genotype while controlling for novelty exposure. Both these conditions caused a significant increase in cFOS staining in all brain regions relative to mice taken directly from the home cage (data not shown).

**Figure 2:**
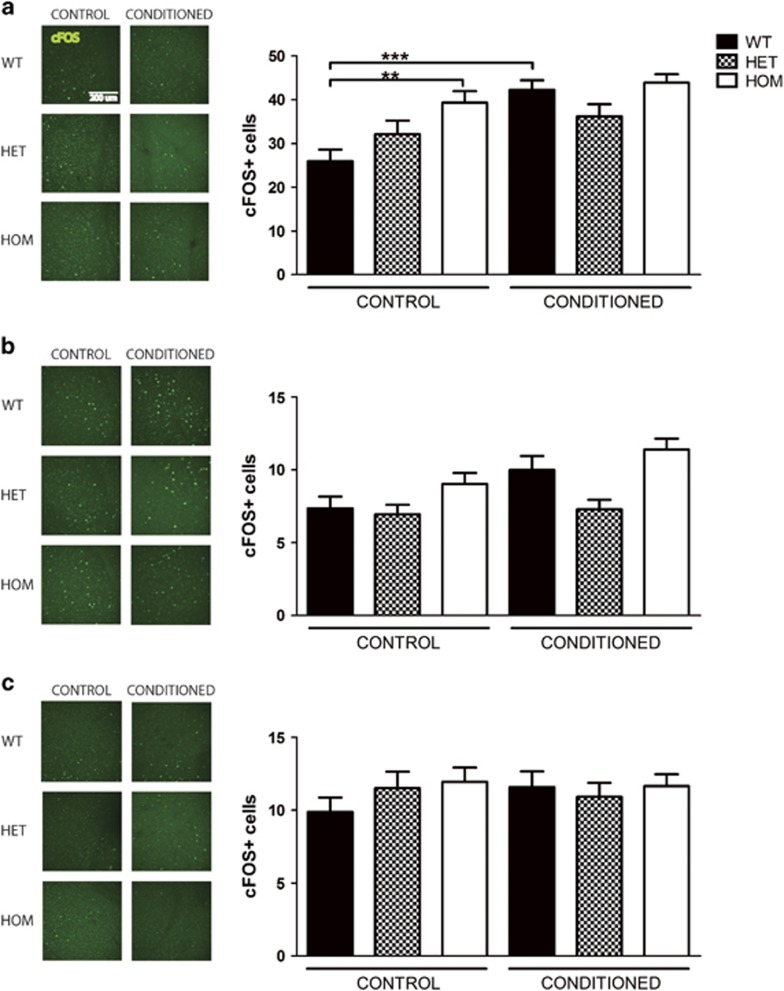
*Rapgef6* deletion impairs amygdala neural activation during fear conditioning. (**a**) Basolateral nucleus of the amygdala cFOS staining was significantly increased in HOM mice over WT at baseline (*P*<0.01). WT cFOS significantly increased after fear conditioning (*P*<0.05), but no other genotype had an increase in cFOS. Scale bar, 200 μm for all micrographs. (**b**) Lateral nucleus cFOS staining was significantly affected by fear conditioning (*P*<0.0001). *Post hoc* comparisons of genotype-specific effects within and between fear conditioning groups were not significant (*P*>0.05). (**c**) Central nucleus cFOS staining was not significantly affected by genotype or fear conditioning. *P<0.05, **P<0.01 and ***P<0.001. HET, heterozygous; HOM, homozygous; WT, wild type.

In WT animals, cFOS expression was significantly affected by conditioning in the basolateral and lateral amygdala but not in the central amygdala (Figures 2a–c, *N*=3 mice and *n*=8 sections per mouse per genotype for all cFOS experiments, ANOVA, basolateral: F_(5,271)_=6.76, *P*<0.0001, lateral: F_(5,272)_=5.29, *P*=0.0001, central: F_(5,265)_=0.57, *P*=0.73). On *post hoc* testing, however, only in the basolateral amygdala was WT cFOS expression significantly increased in fear conditioned as compared with unconditioned WT animals (Bonferroni *post hoc* *P*<0.0001); this was a trend in the lateral amygdala.

**Figure 3:**
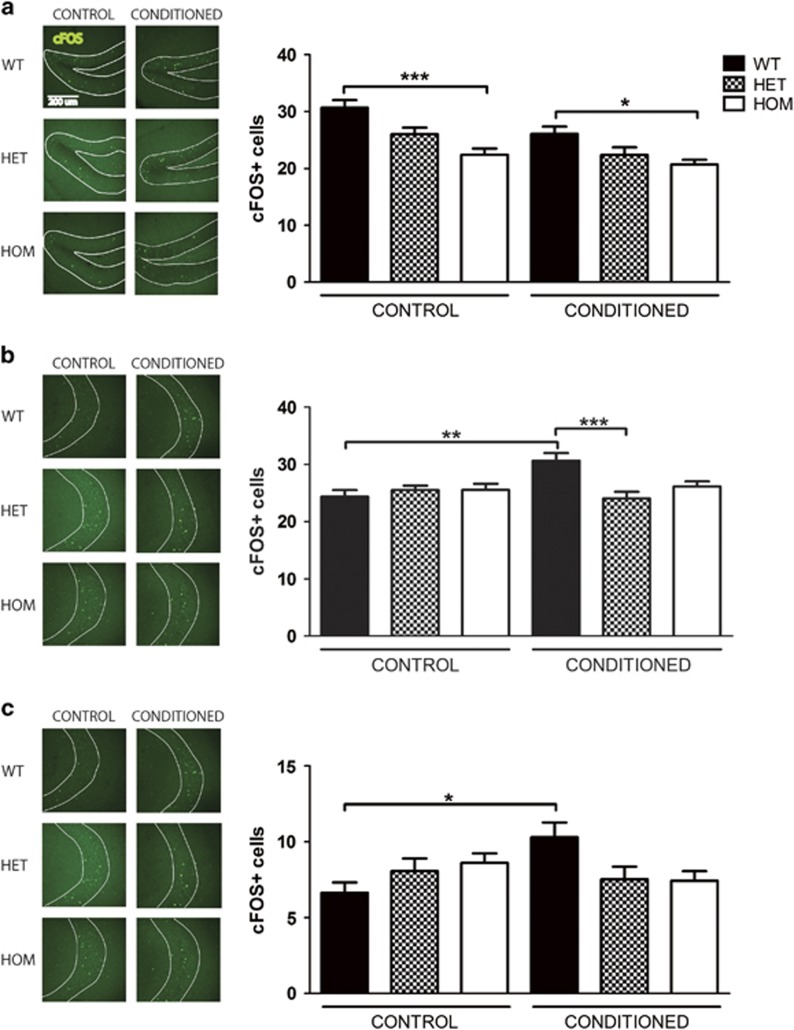
*Rapgef6* deletion impairs hippocampal neural activation during fear conditioning. (**a**) Dentate gyrus subregion of the hippocampus had reduced cFOS staining in HOM mice compared with WT at baseline (*P*<0.01) and after fear conditioning (*P*<0.05). Scale bar, 200 μm for all micrographs. (**b**) CA3 cFOS staining was increased in WT mice after fear conditioning (*P*<0.01) but not HET or HOM mice. (**c**) CA1 cFOS staining was increased in WT mice after fear conditioning (*P*<0.05) but not HET or HOM mice. *P<0.05, **P<0.01 and ***P<0.001. HET, heterozygous; HOM, homozygous; WT, wild type.

In the hippocampus, WT cFOS staining levels in the dentate gyrus, CA3 and CA1 regions were all significantly affected by conditioning (Figures 3a–c, ANOVA, DG: F_(5,222)_=9.13, *P*<0.0001, CA3: F_(5,223)_=4.72, *P*=0.0004, CA1: F_(5,220)_=2.63, *P*=0.02). There was significantly increased cFOS in WT fear-conditioned mice in areas CA3 and CA1 (Bonferroni *post hoc* *P*<0.01, *P*<0.05, respectively vs unconditioned). These results suggest fear conditioning specifically increases cFOS activation in many subnuclei of the amygdala and hippocampus within our protocol.

In contrast, HET and HOM mice failed to significantly increase cFOS expression following fear conditioning in any brain region examined (Bonferroni *post hoc P*>0.05 for all comparisons). As WT cFOS activity increased with fear conditioning but HET and HOM did not, this suggests HET and HOM mice did not adequately activate the amygdala or hippocampus in response to fear conditioning.

Instead, two unusual patterns were observed in knockout mice. In the basolateral amygdala, HOM unconditioned cFOS levels were higher than WT unconditioned, but this pattern did not persist after conditioning (Figure 2a, Bonferroni *post hoc* *P*<0.01). Furthermore, in the dentate gyrus, regardless of conditioning, HOM mice had reduced cFOS activity compared with WT mice, with a downward trend in HET mice (Figure 3a, Bonferroni *post hoc P*<0.0001 unconditioned, *P*<0.05 after fear conditioning). Thus HET and HOM mice had consistently less dentate gyrus activity, but this was unassociated with fear conditioning.

Overall, although WT animals responded to fear conditioning by increasing cFOS activation and thus neural activity in the basolateral amygdala and hippocampal CA3 and CA1, HET and HOM animals did not. Thus *Rapgef6* deletion impaired neural activation in key brain regions associated with fear learning and caused instead an increase in baseline activity in the basolateral amygdala and a decrease in baseline activity in the dentate gyrus.

### Glutamatergic synaptic transmission in the cortico–amygdala projections is normal in *Rapgef6*^*−/−*^ mice

**Figure 4:**
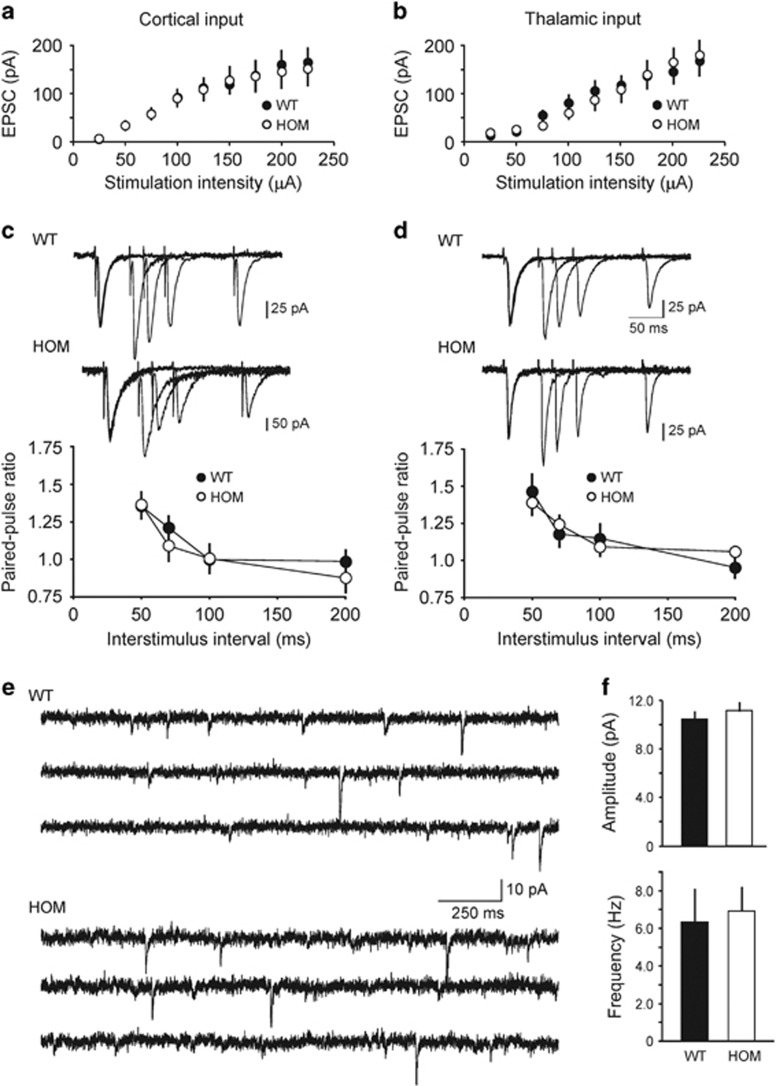
Basal synaptic transmission in the LA is normal in *Rapgef6* knockout mice. (**a**) Synaptic input–output curves for the EPSCs recorded at the cortico-LA synapses in slices from WT and HOM mice. The EPSCs were recorded under voltage-clamp conditions at a holding potential of −70 mV. (**b**) Same as in **a**, but the EPSCs were recorded in thalamic input to the LA. (**c**) Top, examples of paired-pulse facilitation of the cortico–amygdala EPSCs recorded at different interstimulus intervals (50 ms, 70 ms, 100 ms and 200 ms) at a holding potential of −70 mV in slices from WT and HOM mice. Superimposed traces are averages of 10 EPSCs at each interstimulus interval. Bottom, summary plot of paired-pulse facilitation experiments in cortico-LA projections. (**d**) The experiments were identical to **c** but the EPSCs were recorded in thalamic input to the LA. (**e**) *Rapgef6* ablation had no effect on the parameters of glutamatergic mEPSCs. mEPScs were recorded in LA neurons at −70 mV in slices from WT (upper) and HOM mice (lower) in the presence of 1 μM TTX. (**f**) Summary plot showing mean peak amplitude (upper) and frequency (lower) of mEPSCs recorded in LA neurons in slices from WT and HOM mice. Results are shown as mean±s.e.m. EPSC, excitatory postsynaptic current; HET, heterozygous; HOM, homozygous; LA, lateral nucleus of the amygdala; TTX, tetrodotoxin; WT, wild type.

As auditory fear conditioning was impaired in *Rapgef6*^*−/−*^ mice, we explored the effects of *Rapgef6* ablation on excitatory synaptic transmission in projections to the lateral nucleus of the amygdala (LA) from the auditory thalamus and auditory cortex, which deliver the conditioned stimulus (CS) information to the amygdala during both the acquisition and retrieval of conditioned fear memory.^51^ To assay synaptic function in auditory inputs to the LA, we recorded EPSCs in LA principal neurons, stimulating fibers either in the external capsule (cortical input) or the internal capsule (thalamic input).^31, 32, 52, 53^ Notably, synapses in these two converging pathways could be activated independently with our stimulation techniques.^54^ We found that synaptic strength, assayed with synaptic input–output curves for the AMPA receptor-mediated EPSCs, was unaffected in *Rapgef6*^*−/−*^ mice in both studied inputs to the LA (Figures 4a and b; cortical input: *n*=10 neurons from four control mice, *n*=13 neurons from three *Rapgef6*^*−/−*^ mice, two-way ANOVA, F_(1,189)_=0.03, *P*=0.86; thalamic input: *n*=11 neurons from four control mice, *n*=11 neurons from three *Rapgef6*^*−/−*^ mice, two-way ANOVA, F_(1,180)_=0.08, *P*=0.77). The magnitude of paired-pulse facilitation, which, if changed, is indicative of changes in the probability of neurotransmitter release,^31^ was also not different between control and *Rapgef6*^*−/−*^ mice at both cortico-LA and thalamo-LA synapses (Figures 4c and d; cortical input: *n*=13 neurons from six control mice, *n*=8 neurons from three *Rapgef6*^*−/−*^ mice, two-way ANOVA, F_(1,76)_=0.7, *P*=0.41; thalamic input: *n*=13 neurons from four control mice, *n*=9 neurons from three *Rapgef6*^*−/−*^ mice, two-way ANOVA, F_(1,80)_=0.13, *P*=0.72). This finding indicates that *Rapgef6* ablation had no effect on presynaptic function in the CS pathways. Moreover, we found no differences between control and mutant mice in the frequency or amplitude of spontaneous miniature excitatory postsynaptic currents (mEPSCs), recorded in principal neurons in the LA in the presence of a sodium channel blocker tetrodotoxin (1 μM; Figures 4e and f; *n*=15 neurons from four control mice, *n*=9 neurons from three *Rapgef6*^*−/−*^ mice; frequency: unpaired *t*-test, *P*=0.82; amplitude: unpaired *t*-test, *P*=0.49). Taken together, these results show that genetic ablation of the *Rapgef6* gene had no effect on basal excitatory synaptic transmission or synaptic facilitation in the LA.

### Spike timing-dependent LTP in the cortico–amygdala projections is enhanced in *Rapgef6*^*−/−*^ mice

**Figure 5:**
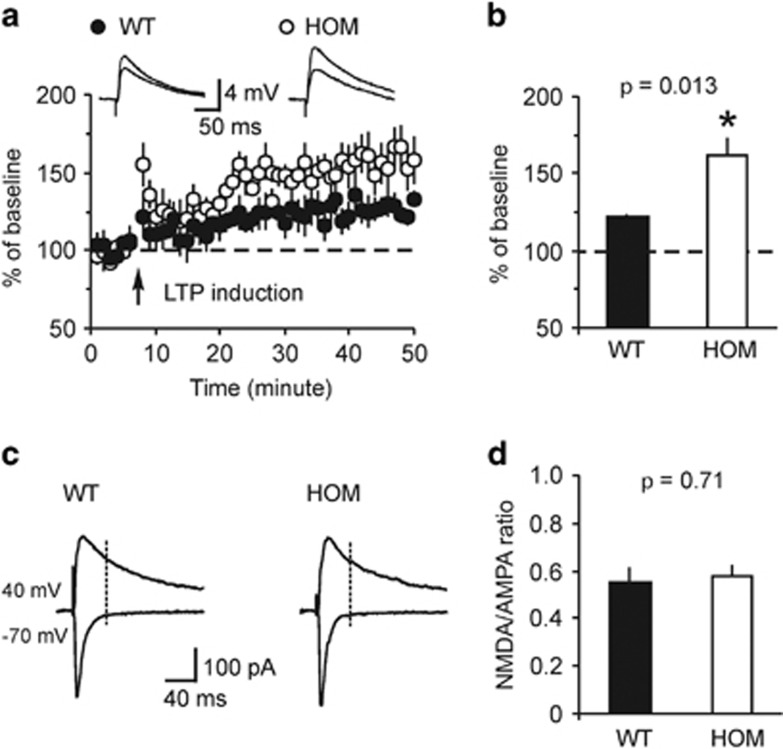
Spike timing-dependent LTP in cortical input to the LA is enhanced in *Rapgef6* knockout mice. (**a**) Spike timing-dependent LTP at the cortico-LA synapses in slices from WT and HOM mice. Insets show the average of 15 EPSPs recorded in current-clamp mode before and 40 min after induction. (**b**) Summary of LTP experiments in cortical input to the LA. (**c**) Evoked cortico-LA EPSCs (average of 15 traces) were recorded sequentially in same neurons at holding potentials −70 mV (bottom) and +40 mV (top) in slices from WT and HOM mice. The NMDA receptor-mediated component of the EPSC was measured at +40 mV at the dashed lines. (**d**) Summary of the NMDA/AMPA ratio values in slices from WT and HOM mice. Results are shown as mean±s.e.m. EPSP, excitatory postsynaptic potential; HOM, homozygous; LA, lateral nucleus of the amygdala; LTP, long-term potentiation; WT, wild type.

Previous studies provide evidence that the mechanisms of LTP in the auditory CS pathways may contribute to the encoding and retention of conditioned fear memory.^31, 55, 56, 57^ Therefore, fear-conditioning deficits observed in *Rapgef6*^*−/−*^ mice could result from LTP impairments in inputs to the LA delivering CS information. To test this possibility, we examined LTP of the EPSPs in cortical input to the LA in slices from control and mutant mice. LTP was induced in current-clamp mode by pairing presynaptic stimuli delivered at 2 Hz with action potentials evoked in a recorded postsynaptic neuron with 4–8 ms delay from the onset of each EPSP in the presence of the GABA_A_ receptor antagonist picrotoxin (50 μM; Figure 5a).^32, 58^ Unexpectedly, we found that the magnitude of spike timing-dependent LTP at the cortico-LA synapses was enhanced in slices from *Rapgef6*^*−/−*^ mice compared with slices from control animals (Figure 5b; *n*=5 neurons from three control mice, *n*=6 neurons from five *Rapgef6*^*−/−*^ mice; unpaired *t*-test, *P*=0.013). The facilitating effect of the *Rapgef6* ablation on LTP was not due to enhancements in the NMDA receptor-mediated synaptic responses, as we did not observe differences in the NMDA/AMPA amplitude ratio in the evoked EPSCs between control and *Rapgef6*^*−/−*^mice (Figures 5c and d; *n*=12 neurons from four control mice, *n*=15 neurons from six *Rapgef6*^*−/−*^ mice; unpaired *t*-test, *P*=0.71). Given that the amplitude of mEPSCs (reflecting sensitivity of postsynaptic AMPA receptors to glutamate) was unaffected by the mutation, the lack of changes in the NMDA/AMPA amplitude ratio indicates that NMDA receptor-mediated synaptic responses remained unchanged in *Rapgef6*^*−/−*^ mice.

## Discussion

Guided by the convergent results of human genetic studies onto the *RAPGEF* family, we used a variety of assays to determine the effects of deleting *Rapgef6*, which is expressed in the hippocampus and amygdala. Behavioral analysis of a mouse modeling *Rapgef6* deletion determined that amygdala function was the most impaired behavioral domain as measured by reduced fear conditioning and anxiolysis. The more disseminated behavioral functions of locomotion and rearing were also increased in the open-field test. Hippocampal-dependent spatial memory was intact in the water maze, as was prefrontal cortex function in a working memory T-maze. *In vivo* neural morphology assessment found CA3 spine density was reduced in knockout animals but additional hippocampal, medial prefrontal cortex and amygdala parameters were unaffected.

These results led us to investigate neural activation as measured by cFOS levels, which demonstrated a reduction in hippocampal and amygdala activation after fear conditioning with baseline activity decreased in the dentate gyrus and increased in the basolateral amygdala. Furthermore, electrophysiological analyses in inputs from the auditory thalamus and cortex to the LA, an essential part of the circuits underlying fear learning, found no effect on pre- or postsynaptic functions but revealed an increase in LTP in knockout brains. Overall, our findings suggest that *Rapgef6* deficiency may lead to functional alterations in amygdalar neural circuitry. Although a link has been established between cFOS expression and long-term synaptic plasticity,^59^ it is, at present, challenging to correlate our cFOS and electrophysiology findings directly. cFOS activation could be affected by frequency of afferent input or alterations in neuronal activity that are not reflected in synaptic plasticity assays we utilized in our study. Alternatively, there may be changes in cell signaling pathways downstream of *Rapgef6* that are independently affecting both cFOS activation and synaptic plasticity.

There are several ways in which these results are analogous to findings from existing Rap and Rapgef family mouse models. A *Rapgef1* hypomorph mouse (due to early embryonic deletion lethality) had reduced neurons because Rapgef1 mediates neural precursor cell cycle exit.^60^ *Rapgef2* knockout caused cortical heterotopia and failure of axonal decussation in the corpus callosum, indicating a role in neural migration and axon guidance.^33, 61^ Both of these phenotypes are far more severe, suggesting *Rapgef6* is involved in alternate downstream pathways. Mutations in *RAPGEF4* were associated with autism,^18^ while *RAPGEF3* single-nucleotide polymorphisms were associated with anxiety and depression.^17^ Individual knockout of either *Rapgef3* or *4* had no behavioral effect while the double knockout led to reduced spatial memory which we did not observe, suggesting there is potential for compensation within this gene family.^62^

Downstream, constitutively active Rap1 *in vitro* recruited Afadin (a *Rapgef6* binding partner) resulting in thinner spines with fewer AMPA receptors.^63^ Cortical *Rap1* deletion caused reduced LTP and increased basal synaptic transmission in the cortico–amygdala but not the thalamo–amygdala pathways.^64^ Similar to our study, Rap1 deletion was associated with impaired cued fear conditioning but normal spatial memory, though we did not find the same reduction in cortico–amygdala plasticity in *Rapgef6*-deficient mice.^64, 65^ Although our behavioral results correlate well with this earlier study, implying that Rap1 may be mediating some downstream aspects of *Rapgef6* function, there are notable differences. Such differences could stem from the fact that the Rap1 knockout is restricted to the cortex only, whereas *Rapgef6* is deleted from the entire brain. This may affect observed functional phenotypes involving non-cortical structures such as the amygdala.

Rap2 may counteract Rap1 by inhibiting spines and increasing synaptic depression as constitutively active forebrain Rap2 led to fewer, shorter CA1 spines with increased long-term depression.^66^ Unlike the *Rapgef6* knockout mouse, constitutively active cortical Rap2 overexpression led to poor spatial learning in the Morris water maze and normal fear conditioning but decreased fear extinction, though the two models share open-field hyperactivity.^66^ Again, direct comparison between transgenic strains and extrapolation from an overexpression model to a knockout model is difficult, and Rap2 deletion, which has not been described, may have different effects than those predicted by currently available data. Overall, deletion of *Rapgef6* partially overlapped phenotypically with *Rap1* deletion, but not as closely with *Rap2* or *Rapgef3/4* models. Despite biochemical predictions, behavioral analysis suggests neural Rapgef6 may be activating Rap1 more than Rap2. Notably, protein analysis of frontal cortex determined that Rap1 levels were reduced in individuals with schizophrenia or depression but not bipolar disorder.^67^

As hippocampal function was normal in spatial testing and the amygdala is necessary for both contextual and cued fear learning, it is likely that amygdala dysfunction could cause the observed fear phenotype. These behavioral results are strengthened by cFOS analysis demonstrating impaired HET and HOM activation in the BLA and hippocampal regions CA3 and CA1 following fear conditioning. Baseline hypoactivity noted as reduced cFOS staining in HOM dentate gyrus suggests that the dentate would be an appropriate region to study as neurogenesis promotes contextual fear conditioning.^27, 68^

Considering all levels of analysis, deletion of *Rapgef6* most significantly impacts the amygdala, a brain region particularly significant for neuropsychiatric disease research. The mouse phenotype may be due to inappropriately elevated LTP at projections from the cortex to the lateral amygdala. Through as yet undetermined mechanisms, baseline cFOS activation was increased in mutant mice in the basolateral amygdala, downstream of the lateral nucleus in fear processing, and there was a failure to recruit this nucleus during fear processing. These findings were associated with altered fear conditioning, either via synaptic alterations or circuit level changes. Interestingly, there are other examples of mouse models, such as the Stathmin knockout, with fear deficits but normal amygdala morphology and baseline neurotransmission in inputs to the LA.^69^

Although LTP is canonically viewed as the neural mechanism of learning, including fear conditioning,^70^ numerous previous studies on genetically modified mice reported impaired spatial or fear learning despite enhanced LTP.^71, 72, 73, 74, 75^ Increased LTP may be functionally suboptimal under certain conditions, as specific levels of potentiation at different components of the circuitry underlying learned behavior might be needed for formation and retention of the memory trace.

As both thalamo-LA and cortico-LA projections are implicated in the acquisition of fear memory to the auditory CS, a resulting behavioral outcome in mutant mice may be determined by the balance between synaptic modifications in these convergent pathways.^46^ Alternatively, the observed dissociation between the effect of *Rapgef6* deletion on LTP in cortical input to the LA and fear conditioning may suggest that the link between LTP in the CS pathways and fear learning is not as straightforward as postulated previously; synaptic plasticity in other parts of the extensive circuitry of fear conditioning could also contribute to the behavioral phenotype.

We present evidence of functional but not anatomical disruption in behavioral circuits. Mechanistically, we hypothesize that Rapgef6 affects Rap1 activity, which has been proposed to suppress cortico–amygdala glutamate release via L-type calcium channel modulation and thus increase the threshold of sensitivity for fear learning.^65^ Mutations in *Rapgef6* enhance LTP at inputs to the amygdala delivering the CS information, possibly leading to nonspecific neural activation during fear learning and impaired behavior on fear-conditioning recall. Altered function of both sides of the synapse is likely contributing to a disease as complex and heterogeneous as schizophrenia. Specifically, there is ample evidence for both presynaptic^76, 77^ and postsynaptic^78^ dysfunction in amygdala and elsewhere in the brain in schizophrenia. Consistent with this, both pre- and postsynaptic forms of LTP co-exist at the amygdalar synapses.^79^

*Rapgef6* was investigated via a mouse model because it was implicated in schizophrenia risk. The change in fear-related behaviors as assessed by the fear-conditioning paradigm is an acknowledged but not commonly studied symptom of schizophrenia that is also relevant to neuropsychiatric disorders such as posttraumatic stress disorder and anxiety disorders.^80, 81^ Moreover, there is growing interest in the role of the amygdala in schizophrenia as some magnetic resonance imaging and functional magnetic resonance imaging studies have demonstrated reduced amygdala volume and function in patients with schizophrenia.^82, 83, 84^ Despite the volumetric findings, postmortem analysis found no changes in volume, neural density or soma size in patients with schizophrenia.^85, 86^ Microarray analysis demonstrated alterations in genes involved in presynaptic function, myelination and signaling, suggesting there may be more subtle dysregulation.^76^ Recently, the mouse model of schizophrenia-associated gene *Tcf4* overexpression was shown to impair trace fear conditioning and reduce cFOS transcription in the anterior cingulate cortex and hippocampus.^87^ On the basis of these findings, it has been suggested that amygdala dysfunction may underlie negative schizophrenia symptoms.^44^

The behavioral phenotype described here supports the utility of *Rapgef6* deletion as a model of neuropsychiatric disease, particularly schizophrenia. This model demonstrated phenotypes associated with schizophrenia including hyperactivity and amygdala dysfunction on fear conditioning and cFOS staining analysis. Reduced anxiety and fear learning could also represent an imbalance in these affective circuits and thus a way to learn more about anxiety-related pathways in a mouse model of diminished responsiveness as opposed to increased fear. This mouse model could be interesting for therapeutic testing and further exploration of behavioral components of schizophrenia and other neuropsychiatric diseases because it is based on human genetics and demonstrates functional phenotypes.
