# Supplementary figures and images for: Deletion of Rapgef6, a candidate schizophrenia susceptibility gene, disrupts amygdala function in mice
Source: Transl Psychiatry. 2015 Jun 9;5(6):e577–. doi: 10.1038/tp.2015.75 (PMC4490285; doi:10.1038/tp.2015.75)

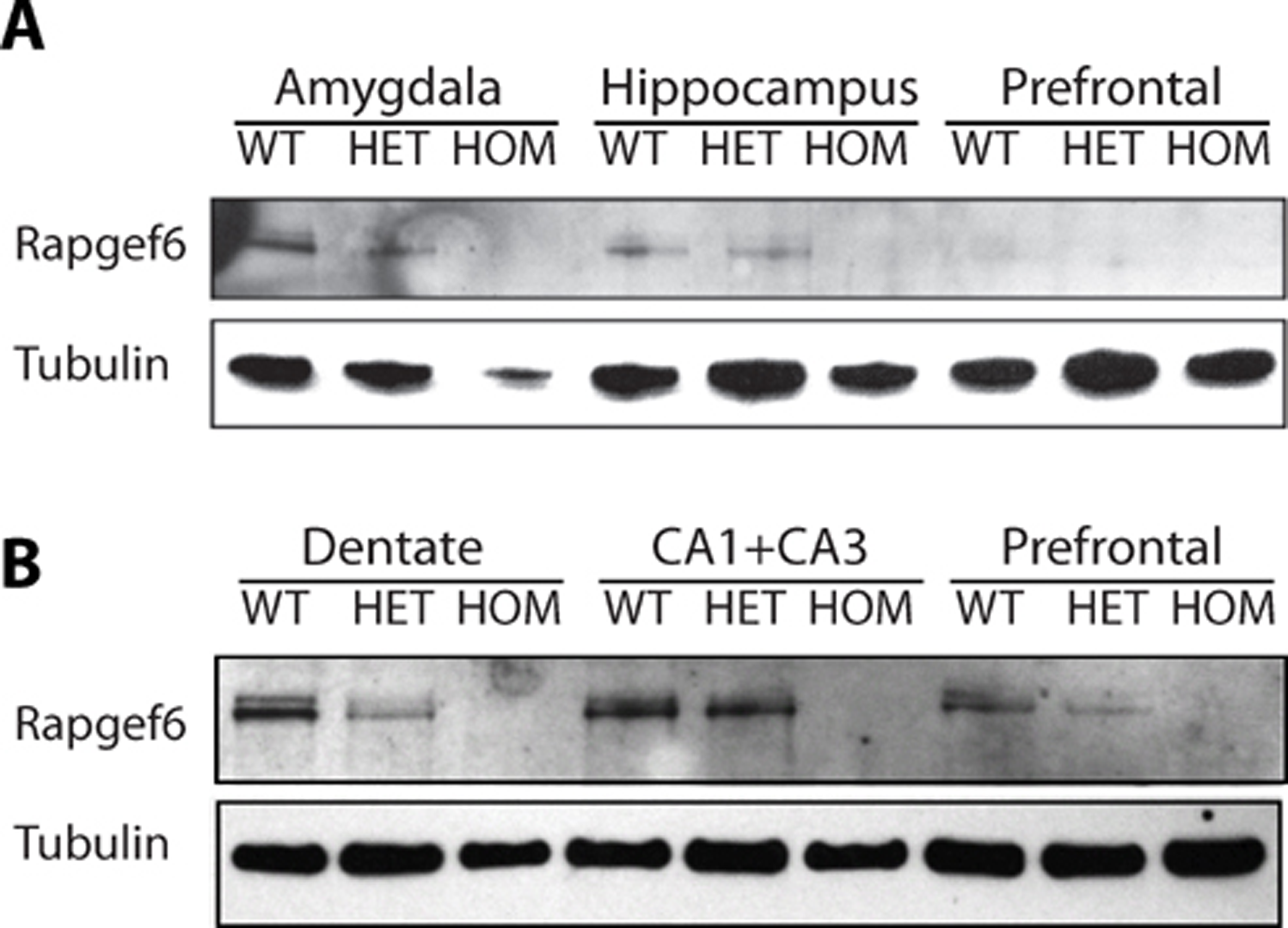

Supplement: Supplementary Figure 1 [file tp201575x1.tif]

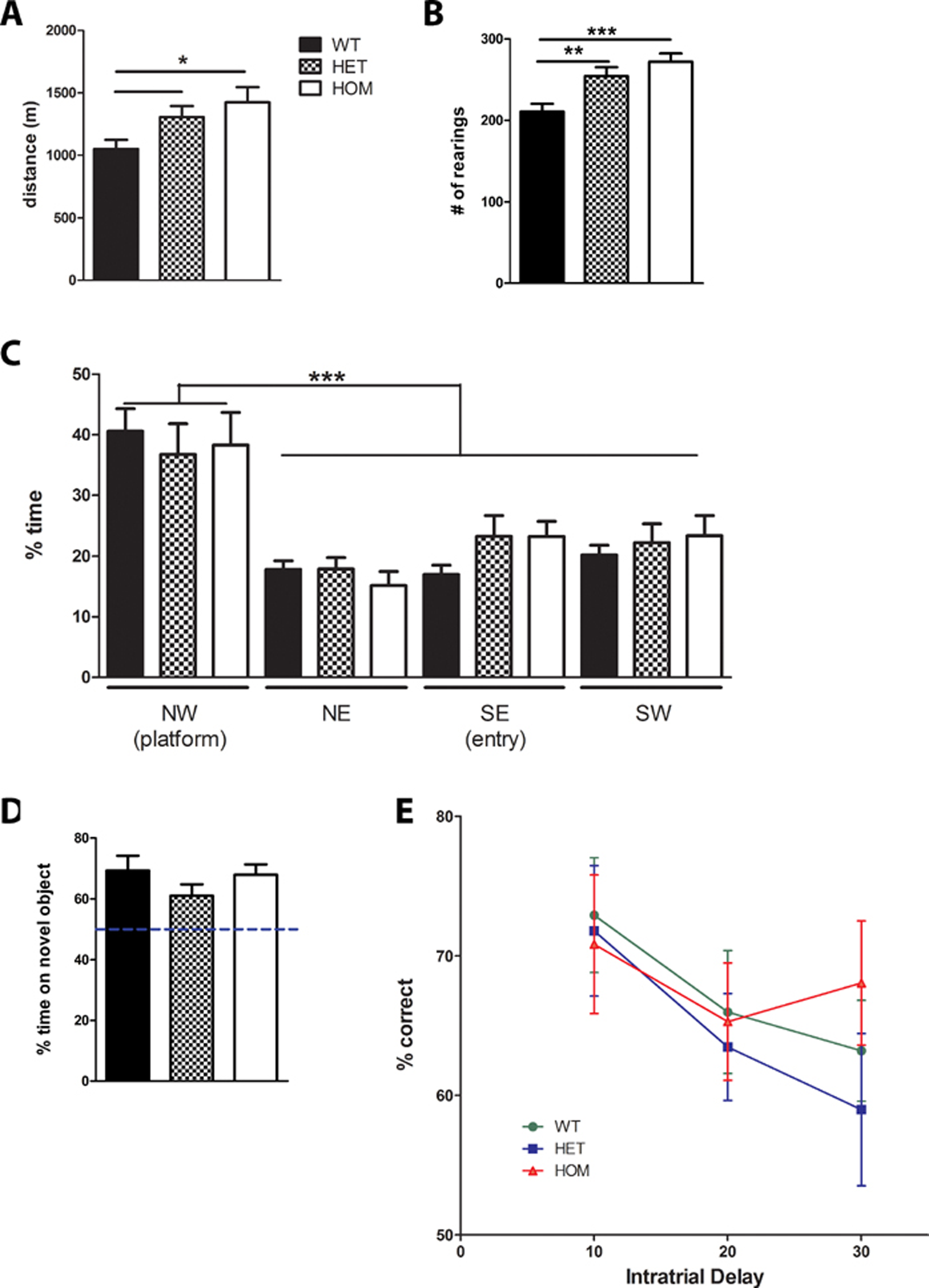

Supplement: Supplementary Figure 2 [file tp201575x2.tif]

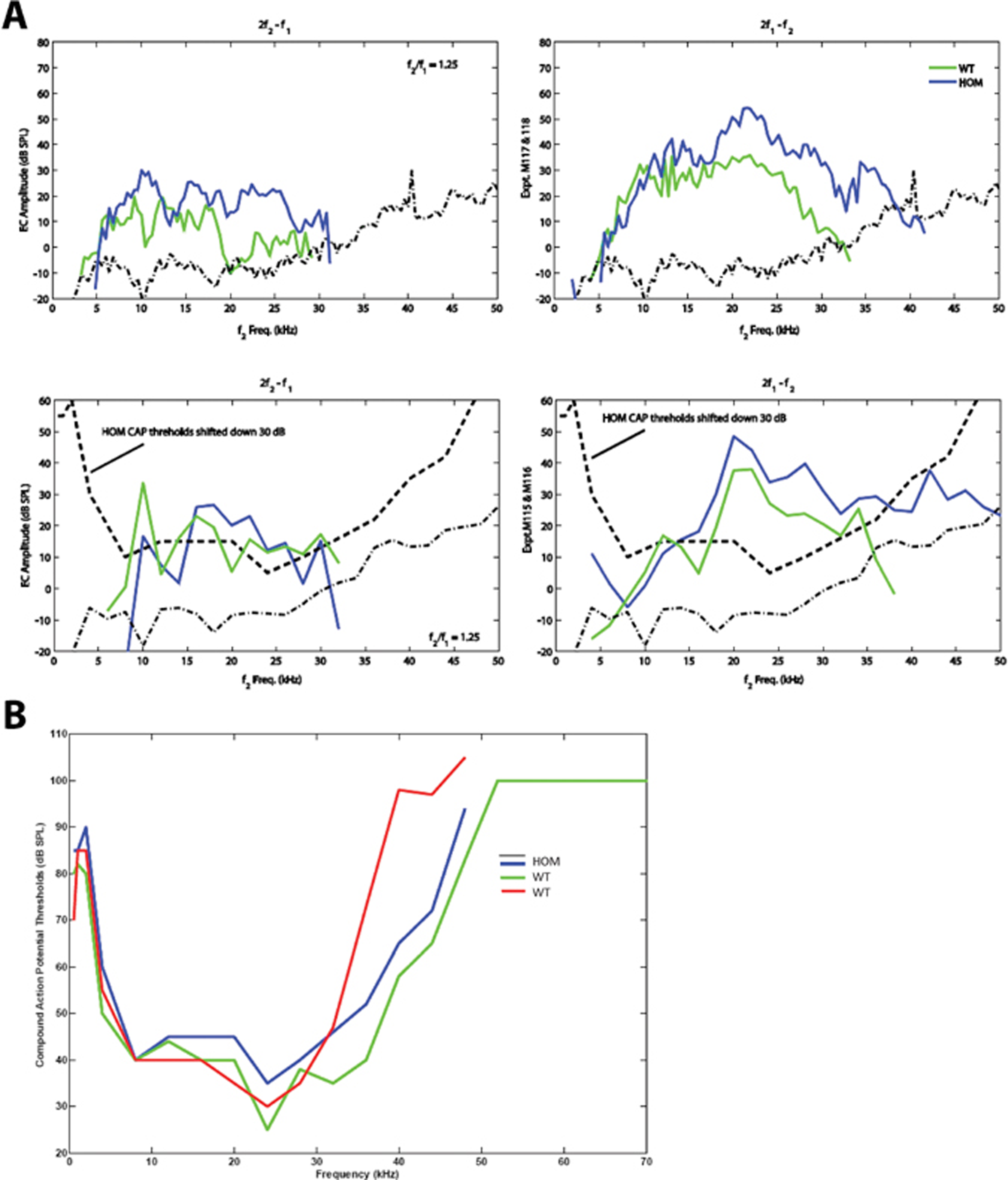

Supplement: Supplementary Figure 4 [file tp201575x4.tif]

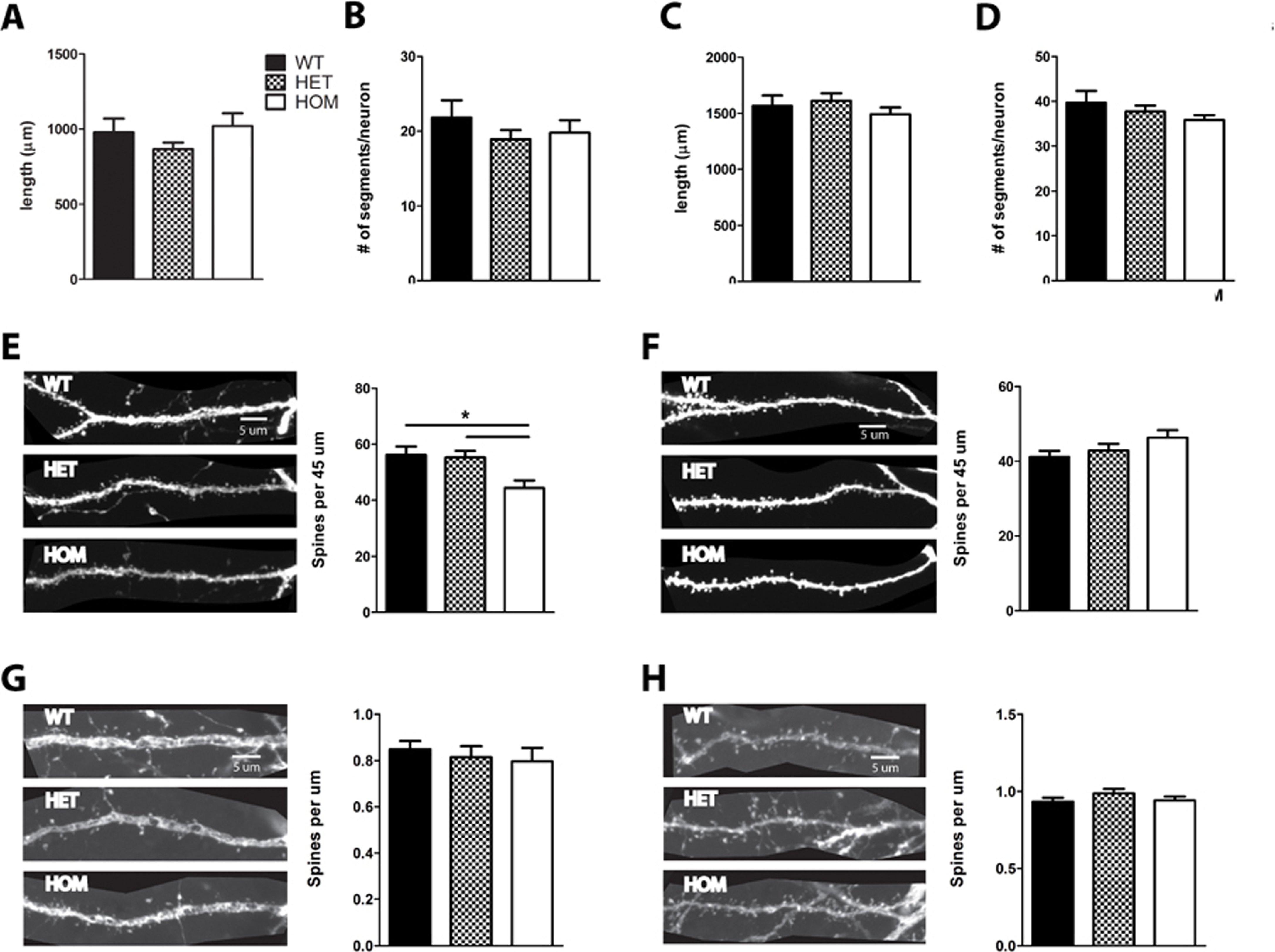

Supplement: Supplementary Figure 5 [file tp201575x5.tif]
