# Supplementary material for: Deletion of Rapgef6, a candidate schizophrenia susceptibility gene, disrupts amygdala function in mice
Source: Transl Psychiatry. 2015 Jun 9;5(6):e577–. doi: 10.1038/tp.2015.75 (PMC4490285; doi:10.1038/tp.2015.75)
Supplement: Supplementary Information [file tp201575x6.doc]

**Supplemental Information**

**Supplemental Figure Legends**

**Supplementary Figure 1.** Rapgef6 protein is expressed in the amygdala and hippocampus.

A) Adult mouse brains demonstrate Rapgef6 protein expression on Western blot in the amygdala and hippocampus more than in the prefrontal cortex.

B) Synaptosomal fractions of adult mouse brains demonstrate Rapgef6 enrichment throughout the hippocampus and the prefrontal cortex.

**Supplementary Figure 2.** Rapgef6 deletion affects open field anxiety but does not alter hippocampal-dependent cognitive tasks.

A) On open field day 2, there was a significant effect of genotype on center distance (*p* = 0.017) with increased center distance in HET and HOM mice (*p* < 0.05). For this and all graphs, data represented as mean ± SEM.

B) Rearing was also significantly affected by genotype (*p* = 0.0003) and increased in HET (*p* < 0.01) and HOM mice (*p* < 0.001).

C) Morris water maze performance was not affected by genotype though all animals spent significantly more time in the platform quadrant at the 1 hour probe trial (*p* < 0.001).

D) Novel object recognition was not affected by genotype though all genotypes investigated the novel object more than chance (*p* = 0.0005, 0.03, 0.01 for WT, HET, HOM).

E) T maze performance was not affected by genotype across increasing intratrial delays though there was a significant effect of intratrial delay (*p* = 0.007).

**Supplementary Figure 3.** Rapgef6 deletion does not affect fear conditioning acquisition.

A) During the initial 3min of fear conditioning acquisition, representing the baseline fear response, there was a significant interaction of genotype and time (Two way ANOVA *F* = 4.176, *p* = 0.0049) with two Bonferroni post-hoc correction comparisons significant. Given the incredibly low rates of freezing in all genotypes, while this difference is significant it is not likely to be meaningful. All data in this figure are shown as mean ± SEM.

B) When condensed across time, there was no significant effect of genotype on freezing during this baseline recording prior to onset of tone shock pairings (ANOVA *p* = 0.27).

C) As an assessment of pain response, the freezing during the first shock was compared however there was no significant effect of genotype (ANOVA *p* = 0.71).

D) There was also no effect of genotype on freezing during the second shock of fear conditioning (ANOVA *p* = 0.36).

E) Schema demonstrating the anatomy of the amygdala and its connectivity. LA: lateral amygdala BLA: basolateral amygdala CE: central amygdala nuclei. Visible anatomic boundaries represented in black, functional boundaries in green, and connections for fear conditioning information processing in red arrows.

**Supplementary Figure 4.** Rapgef6 deletion does not affect auditory function.

A) There was no difference in distortion otoacoustic emissions generated by outer hair cells (n = 2 WT, 2 HOM mice).

B) There was no difference in compound action potential threshold in the cochlear nerve (n = 2 WT, 1 HOM mice).

**Supplementary Figure 5.** Rapgef6 deletion affects hippocampal subregion CA3 neural complexity and spine density, but not amygdala or prefrontal cortex neural architecture.

A) Hippocampal subregion CA3 basal dendritic arbor total length was not significantly affected by genotype (*p* = 0.27).

B) The number of CA3 basal dendritic segments was also not affected by genotype (*p* = 0.48).

C) CA1 basal dendritic arbor total length was not affected by genotype (*p* = 0.49).

D) The number of basal dendritic arbor segments in CA1 did not differ by genotype (*p* = 0.31).

E) CA3 basal dendritic arbor spine density along secondary dendrites were significantly affected by genotype (*p* = 0.007). HOM spine density was significantly lower than WT and HET spine density (*p* < 0.05 for both comparisons). Scale bar 5 µm for all micrographs.

F) CA1 basal dendritic arbor spine density was not affected by genotype (*p* = 0.15).

G) Basolateral amygdala apical dendritic arbor spine density was not affected by genotype (*p* = 0.71).

H) Basolateral amygdala basal dendritic arbor spine density was also unaffected (*p* = 0.46).

**Supplementary Table 1. Role of RAPGEF family members in neural function and neuropsychiatric disease.**

|  | **Implicated in** | **Mouse Knockout Neural Phenotype** | **Human**  **Disease** |
| --- | --- | --- | --- |
| **RAPGEF1** | Neuroepithelial cell cycle via b-catenin, cortical migration via radial glia attachment and Reelin, neurite outgrowth | Hyperproliferation of neuroepithelium, reduction in neurons |  |
| **RAPGEF2** | Neurite outgrowth, neural migration, midline axon crossing | Cortical heterotopia, failed commissures | Schizophrenia |
| **RAPGEF3 RAPGEF4** | Long term potentiation and depression, hippocampal function | Double knockout reduces long term potentiation, spatial memory, social interaction via miR124 | Depression  Autism |
| **RAPGEF5** | Downregulated in neuronal differentiation |  |  |
| **RAPGEF6** | Integrin-mediated cell adhesion; epithelial E-cadherin tight junction maintenance; cell migration | Grossly normal, splenomegaly | Schizophrenia |

**Supplemental Experimental Procedures**

*Western blotting*

Crude synaptosomal preparations were made by homogenizing in buffer containing 5 mM Hepes/10% sucrose (pH 7.5). Homogenates were spun down at 1000 × g, and the supernatant was further centrifuged at 12,000 × g. The pellet was resuspended in the same buffer and the protein concentration was determined using a DC protein assay kit (BioRad, Hercules, CA, USA). The protein homogenate was mixed with NuPage LDS loading dye (Invitrogen, ThermoFisher, Waltham, MA, USA) with reducing reagent and boiled for 10 minutes, then loaded onto a 4-12% gradient polyacrylamide gel and run and transferred onto a nitrocellulose membrane according to the manufacturer’s instructions (Invitrogen).

*Novel object recognition*

Mice were habituated for two days in an empty cage 23x46cm. On day three, the cage held two identical green plastic objects affixed at either end and mice were videotaped for 5 minutes. One hour later, they were returned to the cage for 5 minutes with one green object replaced with a yellow plastic object, balanced across genotypes for left and right sides. Three hours later, the novel object was replaced again with a red plastic object. All objects were distinct in texture, shape, and color.

*Morris Water Maze*

In the initial habituation, mice were placed in a 3 gallon bucket of room temperature water with a plastic platform and timed until they climbed onto the platform, twice a day for 3 days. Animals were warmed on a thermoregulated heating pad. Next the platform was marked by a colored pole and then submerged in a 1.7m diameter pool of room temperature water made opaque with non-toxic white paint. The room walls were uniquely decorated to provide extra-maze cues and the tester was concealed by curtains. Mice entered the pool at 8 randomly chosen points using 3 pathways from the home cage to prevent association with entry or tester location, however the platform never moved. Three times a day for two days, mice were manually timed and automatically tracked with AnyMaze Software (Stoelting) until they located the visible platform and remained on it for 30 seconds. In the hidden platform training, the platform marker was removed and animals were timed and tracked until they located the unmarked platform and remained for 30 seconds. After 3 trials per day for 5 days of hidden platform testing, the two probe trials occurred. One hour and 24 hours after the last hidden trial, the platform was removed and mice were automatically tracked for one minute.

*T maze*

Animals were food restricted to 80-90% of their weight. The T maze apparatus has one start arm 38x10cm with two choice arms 26cm long containing 3cm dishes affixed at the end of each arm and is contained by walls 12cm high. Sliding doors separated the arms. Extra food pellets (BioServ, Flemington, NJ, USA) were placed beyond each choice arm in order to mask olfactory cues.

In forced alternation training, mice were allowed to explore one open arm which did not have food then returned to the start zone. During a 5 second intratrial delay this arm was closed off and the opposite goal arm opened and baited with a food pellet. Mice were allowed to explore the goal arm and eat before returning to the start zone. This was repeated 10 times with an intertrial delay of 40 seconds.

In choice training, the goal arm of the maze containing a pellet was initially closed. Each mouse was timed until it reached the empty food dish of the open arm then returned to the start zone for 5 seconds. The center area of the maze was wiped with 70% ethanol to remove olfactory cues and both arms were opened. The mouse was released and the choice of arms (goal or non-goal) and time to the food dish were recorded. Once the animal’s body entered one arm, the door to the other arm was closed. There was a 40 second intertrial delay. This was repeated 10 times daily, pseudorandomized for the goal arm. If mice developed a strong turn bias, goal arms were biased toward the avoided side and 5 extra trials were added until the bias was corrected.

*Fear Conditioning*

On the first day of fear conditioning, animals were habituated then placed into sound-attenuated fear conditioning chambers (Med Associates, St Albans, VT, USA) and videotaped and monitored via FreezeFrame software (Coulbourn). Lemon extract was placed inside each chamber to create an odor. After 3 minutes of habituation, a tone was delivered then a 0.7mA shock was delivered for 2 seconds immediately after the tone. This pairing of tone and shock was repeated 1 minute later.

Twenty-four hours after conditioning, animals were re-exposed to the same context. They were habituated in the same cages, placed into the same chambers with lemon scent, and tracked for 6 minutes. Two hours later, animals were tested for cued conditioning. In order to create a novel context, they were habituated in different cages then placed into the chambers but with plastic pink colored floor and wall inserts and vanilla scent. After 3 minutes in this novel environment the tone played for 3 minutes. Percent time spent freezing was automatically scored by FreezeFrame after the freezing/motion threshold was manually set for each animal on each trial.

*Electrophysiology*

Cold cutting solution (in mM): 252 sucrose, 2.5 KCl, 5.0 MgCl_2_, 1.0 CaCl_2_, 1.25 NaH_2_PO_4_, 26 NaHCO_3_ and 10 glucose, and equilibrated with 95% O_2_ and 5% CO_2_.

Recording solution (in mM): 119 NaCl, 2.5 KCl, 2.5 CaCl_2_, 1.0 MgSO_4_, 1.25 NaH_2_PO_4_, 26 NaHCO_3_, 10 glucose and 0.05 picrotoxin and equilibrated with 95% O_2_ and 5%CO_2_ (pH 7.3–7.4) at 22°C. In current-clamp experiments, the recording patch electrodes (3-4 MΩ resistance) contained (in mM): 135 K-gluconate, 5 NaCl, 1 MgCl_2_, 0.2 EGTA, 10 HEPES, 2 MgATP, and 0.1 NaGTP (adjusted to pH 7.2 with KOH). In voltage-clamp experiments, 135 mM Cs-methane-sulfonate was used instead of potassium gluconate.

*Auditory testing*

Two pairs of WT and HOM 5-7 week old female animals underwent auditory testing. Mice were anesthetized with ketamine (13 mg/kg) and urethane (1.5 mg/g). The analgesic buprenorphine (0.1 mg/kg) was also administered for maintenance of anesthesia. At the end of the experiment, the animal was sacrificed with an overdose of urethane or sodium pentobarbital. During the experiment, animal body temperature was maintained at ~ 37° C using a thermostatically controlled heating blanket. A tracheotomy was performed to maintain a patent airway. The left pinna was removed. To measure the compound action potential (CAP) thresholds, the bulla was opened with great care.

Acoustic stimuli were generated and collected digitally using Tucker Davis Technologies System III (TDT; Alachua, FL, USA). Stimulus and acquisition programs were written in Matlab and TDT Visual Design Studio. The sampling frequency of the TDT system was 200 kHz. Data were stored following removal of the first 4096 points of the response waveform to avoid the transient, and time-averaging the remaining waveform, typically in 50 time-locked segments. Responses were later analyzed by Fourier transform in Matlab.

The ear was acoustically stimulated via a 40 – 1377 tweeter (Radio Shack). The tweeter and a probe-tube microphone (Bruel and Kjaer model 4134) were coupled together via a T tube and coupled to the ear canal. The probe-tube microphone served as the ear canal pressure monitor. The transfer function of the probe-tube microphone was accounted for when setting the sound pressure level (SPL, decibels relative to 20 Pa peak) and analyzing the data. With a 1 second data acquisition time, the microphone noise level (with probe-tube) was ~ -10 to 25 dB SPL up to 50 kHz. The noise level was determined by the average FFT value of the six adjunct points at frequencies below and above the stimulus. The level of distortion products produced by the system (mainly the driver) has been discussed previously.^1, 2^ With current settings, system distortion was ~ 60 dB smaller than the 80 dB SPL primaries. Therefore, system distortion was not a concern in the results. System distortion was also checked with postmortem responses at the end of each experiment.

Sound-evoked CAP recordings (.5 to 80 kHz) were made from a silver wire electrode firmly connected to the cochlear bony shell at the round window. The reference electrode was connected to the neck muscle and the animal was grounded. To get the CAP waveform, anti-phase single tone (1 second duration) stimulus were used and averaged 30 times to remove the cochlear microphonic responses. 5 uV peak-to-peak criteria was used to determine the threshold sound pressure level to each stimulation.

Two equal-intensity tones (1-2s duration) with fixed f_2_/f_1_ = 1.05 or 1.25 were used in DPOAE measurements. The primary frequencies were swept from 1 to 60 kHz in 500 Hz steps.

**Supplemental References**

1. Dong W, Olson ES. Middle ear forward and reverse transmission in gerbil. *J Neurophysiol* 2006; **95**(5)**:** 2951-2961.

2. Dong W, Olson ES. Supporting evidence for reverse cochlear traveling waves. *J Acoust Soc Am* 2008; **123**(1)**:** 222-240.
